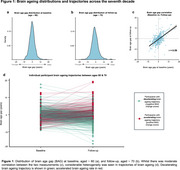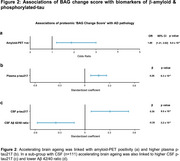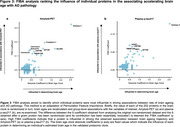# Divergent trajectories of brain ageing across the seventh decade predict AD pathology in a UK population‐based birth cohort

**DOI:** 10.1002/alz70856_100995

**Published:** 2025-12-24

**Authors:** James Groves, Hamilton Se‐Hwee Oh, Amelia Farinas, Veronica Augustina Bot, Dylan M Williams, Andrew Wong, William Coath, Ashvini Keshavan, Patricia Moran‐Losada, Sarah‐Naomi James, Marcus Richards, Jennifer M Nicholas, Alina Isakova, Tony Wyss‐Coray, Jonathan M Schott

**Affiliations:** ^1^ Dementia Research Centre, UCL Queen Square Institute of Neurology, University College London, London, United Kingdom; ^2^ Wu Tsai Neurosciences Institute, Stanford University, Stanford, CA, USA; ^3^ The Phil and Penny Knight Initiative for Brain Resilience, Stanford University, Stanford, CA, USA; ^4^ Medical Research Council Unit for Lifelong Health and Ageing at UCL, London, United Kingdom; ^5^ Stanford University School of Medicine, Stanford, CA, USA; ^6^ Department of Medical Statistics, London School of Hygiene and Tropical Medicine, London, United Kingdom; ^7^ Department of Neurology and Neurological Sciences, Stanford University School of Medicine, Stanford, CA, USA; ^8^ Department of Bioengineering, Stanford University, Stanford, CA, USA

## Abstract

**Background:**

Ageing is the strongest risk factor for Alzheimer's disease (AD), but the molecular mechanisms underpinning this link are not established. In an identically aged birth cohort, we: 1) tracked proteomic brain ageing trajectories across the seventh decade; 2) tested associations between ageing trajectories and AD pathology; 3) identified which individual proteins influenced associations.

**Methods:**

*n* = 414 from the 1946 British Birth Cohort had plasma assayed with SomaScan 11k v5 at baseline (age=63.4±1.1yr) and follow‐up (70.6±0.7yr). Brain age was estimated from a proteomic clock using 202 brain‐enriched proteins. ‘Brain age gap’ (BAG) quantified whether a participant's brain age estimate was older (positive) or younger (negative) than their chronological age. ‘BAG change score’ [ (BAG at follow‐up) – (BAG at baseline) ], reflected accelerating (positive) or decelerating (negative) trajectory. Primary outcomes were amyloid‐PET positivity (CL cut‐point=11.9) and log‐transformed plasma *p*‐tau217 (ALZpath SIMOA). Secondary outcomes in a subset (*n* = 114, 72.9±0.59yr) were log‐transformed CSF *p*‐tau217 and Aβ42/40 ratio. We used linear/logistic regression for continuous/binary outcomes, adjusted for sex. Individual protein analysis used Feature Importance for Biological Ageing (FIBA).

**Results:**

BAG at baseline ranged from ‐9.1 to +17.6yrs. Even greater divergence was seen at follow‐up (range: ‐16.93, 19.16 years). Whilst measurements were correlated (r=0.59), we observed heterogenous brain ageing trajectories across the seventh decade (BAG change score range: ‐21.3 to 17.3 years, Figure 1). Baseline BAG did not predict biomarker outcomes some ∼10yr later. BAG at follow‐up associated with higher plasma *p*‐tau217 (b=0.18, *p* = 1.4x10^‐4^) but not amyloid‐PET status. Higher BAG change score (i.e., accelerating brain ageing) was associated with amyloid‐PET positivity (OR 1.89, *p* = 6.4x10^‐3^, Figure 2), higher plasma *p*‐tau217 (b=0.26, *p* = 6.2x10^‐4^), higher CSF *p*‐tau217 (b=0.30, *p* = 9.5x10^‐3^) and lower CSF Aβ 42/40 ratio (b=‐0.35, *p* = 2.2 x10^‐3^). Associations remained significant adjusting for APOE_4_ status, plasma neurofilament light and GFAP. Influential proteins in associations included Aldolase C, NPTXR and LRRTM2 (Figure 3).

**Conclusion:**

An identically‐aged population experienced heterogenous trajectories of proteomic brain ageing across their seventh decade. Accelerating brain ageing was predictive of AD biomarker positivity, independent of non‐specific measures of neurodegeneration and genetic risk. Future research will explore how implicated proteins may couple brain ageing to AD pathology.